# Identification of organizational barriers to HPV vaccination uptake in medical students in southern Italy: a cross-sectional study

**DOI:** 10.3389/fpubh.2023.1272630

**Published:** 2023-11-17

**Authors:** Michele Sorrentino, Michelangelo Mercogliano, Federica Esposito, Agostina Maria Lamberti, Gaetano Buonocore, Carla Riganti, Maria Triassi, Raffaele Palladino

**Affiliations:** ^1^Department of Public Health, University “Federico II” of Naples, Naples, Italy; ^2^Clinical Directorate, niversity Hospital “Federico II” of Naples, Naples, Italy; ^3^Interdepartmental Research Center in Healthcare Management and Innovation in Healthcare (CIRMIS), Naples, Italy; ^4^Department of Primary Care and Public Health, School of Public Health, Imperial College, London, United Kingdom

**Keywords:** HPV, vaccine, barriers, questionnaire, knowledge, attitude, medical students

## Abstract

**Introduction:**

Despite proven efficacy, HPV vaccination coverage is still suboptimal. Factors influencing vaccination uptake are education attainment, socio-economic position, and knowledge about HPV. This study aimed to assess HPV vaccination uptake and its correlates among medical students and identify logistic-organizational barriers, knowledge, and attitudes with regard towards HPV vaccination to improve current public health vaccination strategies. Medical students, with their acquired biological knowledge, were selected as a low-risk groups for HPV vaccination uptake. This cross-sectional study was conducted using a validated questionnaire.

**Methods:**

Students in their the first 3 years of study students were preferentially invited. Eventually, the invitation was extended to every medical student. Logistic multivariable regression was used to assess determinants of HPV vaccination uptake. Additional analysis explored determinants of knowledge of and attitude toward HPV vaccination. Finally, a sensitive analysis was conducted to further assess the effect of knowledge and attitude on the HPV vaccination rate.

**Results:**

A total of 882 medical students participated, with 74.5% enrolled in the first 3 years of their training. HPV vaccination uptake was 55.5%, ranging from 78.5% for females to 16.5% for males. Male sex and increasing age were consistently associated with a lower vaccination uptake (males sex: OR 0.03, CI 0.02–0.05; age: OR 0.77, CI 0.68–0.88), whereasilst progress in their academic career was associated with a to higher likelihood of being vaccinated (6th year: OR 3.45, CI 1.24–9.57). These associations were confirmed when considering the knowledge of and attitude towards HPV. Additionally also, an active outreach from healthcare institutions was associated with a higher likelihood of receiving HPV vaccination (OR 1.70, CI 1.09–2.65.

**Conclusion:**

HPV vaccination in medical students was higher than in the general population; however, it was still suboptimal. An active and up-to-date call strategy and extending the free-of-charge offer are essential measures for to improvinge vaccination uptake. The findings support the need to improve public health strategies and increase awareness and knowledge ofregarding HPV vaccination.

## Introduction

Human papillomavirus (HPV) is responsible for the most prevalent sexually transmitted infection (1), with a lifetime probability of acquiring it ranging between 85% for females and 91% for males (2, 3). Although HPV primarily causes cervical cancer in females, it also serves as a risk factor for cancers in both sexes (vaginal, vulvar., oropharyngeal, penile, and anal), with some types being more common in males (4), affecting especially vulnerable subgroups (5) and influenced by a large variety of factors (6) (e.g., genotype, gender, anatomic site of infection, and socio-economic position). The burden of HPV-related cancers on health systems is disproportionate as it accounts for 5% of new cancers globally (7). In Europe alone, 58,169 new HPV-related cancers are estimated to occur each year (8). Furthermore, 17,486 women die from cervical cancer every year (1,589 in Italy) (9). Additionally, cervical cancer results in a loss of 436259.6 disability-adjusted life years in the European Union and 35796.71 in Italy in 2019 (10). In 2018, the total annual direct cost of HPV-related disease to the Italian Health System was €542.7 million, with a range of €346.7–€782.0 million (11). These costs could further increase when innovative therapies for cancer treatment are taken into account (range €16.2–€37.5 million) (11).

HPV vaccination is an effective primary prevention strategy to reduce HPV cancer-related infections (12). Vaccines against HPV have been available since 2006 and recommended by the World Health Organization (WHO) since 2009 (13). They have been progressively introduced in many national immunization schedules, initially offered only to female adolescents. However, both vaccine introduction and coverage are still suboptimal (13). It was reported that HPV immunization programs targeted only 20% of young adolescent females worldwide, and only 15% of girls aged 10–20 had been fully vaccinated by the end of 2019 (13). The Italian National Health Service provides the HPV vaccine free of charge to adolescents aged 11–12, with a catch-up dose for 25-year-old females (14). Despite this recommendation, limited success has been reported as the full HPV immunization rates remain far below the WHO threshold of 95% (15).

Barriers to HPV vaccine promotion and uptake are low education, low income, fears of embarrassment or stigma, lack of knowledge about HPV, cultural acceptability of sexual behavior, distrust of healthcare, general lack of information regarding cancer risks and prevention, privacy concerns, and limited access to health care providers and preventive care (16). Other studies have suggested that low vaccination rates may be associated with factors such as parental approval of vaccination (17, 18) and physician recommendations (18–20).

As education attainment, socio-economic position, and knowledge about HPV are among the main factors that can explain the HPV vaccination uptake, it is pivotal to assess whether the coverage effectively increases in groups that are considered at low risk to further improve the public health strategy. Specifically for this study, we selected medical students as a low-risk group for HPV vaccination uptake due to their previously acquired biological knowledge necessary to pass the admission test. This study aims to assess HPV vaccination uptake and its correlates among medical students at the University “Federico II” in Naples, the largest university in Southern Italy, and identify logistic-organizational barriers to, knowledge of, and attitude toward HPV vaccination to improve current public health vaccination strategies.

## Methods

### Study design

This cross-sectional study was conducted between April and May 2023. Data were collected through the administration of a validated anonymous questionnaire. All Italian medical students at the University Hospital “Federico II” of Naples, the largest university hospital in Southern Italy, were invited to participate through multiple communication channels, including email, posters distributed across university premises, and info points. All prospective participants received comprehensive information concerning the research’s purpose and scope, the methodology employed, the voluntary nature of their involvement, the assurance of complete anonymity and confidentiality of their shared information, and the option to discontinue their participation at any time without the need to provide a rationale. Before completing the survey, written consent was secured from each participant. Consent was collected separately from the survey to protect the privacy of the participants. Sample calculation was based on the population size of students attending the first 3 years of medical school, as they were our primary target population (e.g., more likely to have been offered the vaccination free of charge). However, we also extended the possibility to participate in the study to the other medical students. The desired sample size was 843 subjects, considering a population of 3,999 medical students, a confidence level of 95%, and a margin of error of ±3%. Ethical approval was granted by the University Hospital Ethics Committee (Prot. no. 0014525, 24th of March 2023); the study was conducted in accordance with good clinical practice and the Declaration of Helsinki.

### Study variables

Study variables were retrieved from a questionnaire that was adapted from a previously validated questionnaire (21). Before the questionnaire was administered to our target population, it was discussed by a focus group composed of medical doctors and other healthcare workers to evaluate its comprehensibility and intelligibility. The questionnaire is available in Supplementary materials. Study variables included the following socio-demographic characteristics: age, sex (male or female), years of enrollment, and smoking habits (smoker, non-smoker, or former smoker); there was an additional question regarding HPV vaccination status and healthcare-related and organization factors possibly associated with vaccination status (city of residence, having received an invitation to undergo vaccination, and gynecological visits). Attitude was measured using 17 questions, which presented five potential answers (coded as follows: 1 = completely disagree, 5 = completely agree). The average score was obtained by averaging the score of each question. Knowledge was measured using 23 questions, and the possible answers were true or false (coded as follows: 1 for the correct answer or 0). The average score was obtained as a percentage of correct answers, ranging from 0 to 100. Those with missing data for at least three questions were excluded from the analysis. Sexuality information was collected, including the frequency of sexual intercourse over the last year and the use of sexual protection.

### Statistical analyses

Study population characteristics were analyzed using descriptive statistics, as appropriate. Analyses were performed for the entire study population but were also restricted to female students and the sexually active population. Multivariable logistic regression models were employed to identify factors associated with HPV uptake, whereas factors associated with knowledge were explored through multivariable linear regression models and factors associated with attitude were analyzed using multinomial logistic regression models. Models were controlled for sex, age, year of enrollment, smoking habits, province of residence, receipt of an invitation to undergo vaccination (HPV invitation), number of sexual partners over the last year, an ob-gyn visit (only in the female sample), and the use of condoms (only in the sexually active sample). To better assess the contribution of each variable, multiple models were used (presented in Supplementary materials) that progressively adjusted for socio-demographic characteristics (Model I), healthcare-related and organization factors (Model II), and sexuality (Model III). To further explore the correlation of HPV vaccination uptake, we conducted a sensitivity analysis by further adjusting statistical models for knowledge and attitude. The results are presented as odds ratios (OR), regression coefficient (coef.), and 95% confidence intervals (95% CI), as appropriate, and were considered significant if the *p*-value was <0.05. Statistical analyses were performed using Stata MP 15.0 statistical software.

## Results

### Demographic characteristics

Over the study period, 882 questionnaires were completed. Demographic characteristics of the sample are presented in Table 1. Of the sample, 62.9% were female (ages ranged from 18 to 38 years; mean age was 21.6 ± 2.6). More than a third of the sample (34.8%) attended their first year of medical school and 18.6% and 20.9% were enrolled in the second and third years, respectively. Of the sample, 74.3% never smoked and 18.9% were current smokers. In terms of residence, 74.3% of the sample lived in the Naples province. HPV vaccination uptake was 55.5% in our study population, with uptake ranging from 78.5% for females to 16.5% for males.

**Table 1:** Characteristics of the study population.

Study population		*N*	Percentage
**Sample size**		882	
Sex			
Male		325	37.14
Female		550	62.86
Smoking habit			
No		602	74.32
Former smoker		55	6.79
Yes		153	18.89
Year of enrollment			
1st		302	34.87
2nd		161	18.59
3rd		181	20.90
4th		57	6.58
5th		59	6.81
6th		57	6.58
O.P.Y.		49	5.66
Vaccination status			
No		388 (F 110)	44.50
Yes		484 (F 439)	55.50
Province of residence			
Naples		644	74.28
Other		223	25.72
**Age**	Mean	21.56 ± 2.63	

### Main analysis

**Figure 1:**
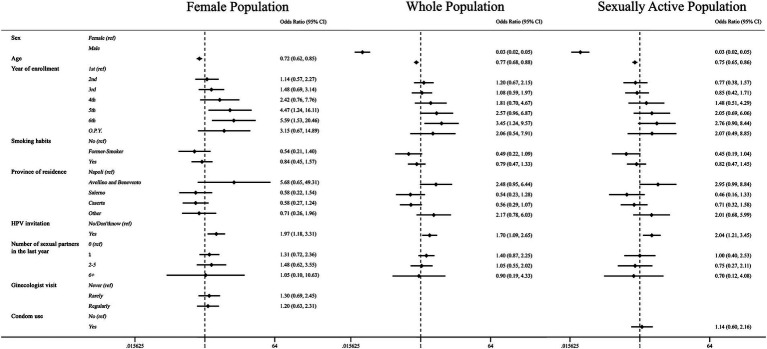
Association between HPV vaccination and demographic characteristics, healthcare-related and organization factors, and sexuality. Multivariate logistic regressions were employed for the whole population (central column), female population (left column), and sexually active population (right column), including HPV vaccination as an outcome variable, and controlled for the following variables: age, gender, year of enrollment, smoking habits, province of residency, received an invitation to undergo vaccination (HPV invitation), number of sexual partners over the last year, obi-gyn visit (only in the female population), and the use of condoms (only in the sexually active population). Results are presented as odds ratios (OR) and 95% confidence intervals (95% CI).

When considering the whole study population, we found that increasing age and male sex were associated with a 23 and 97% reduction in the likelihood of being vaccinated, respectively (age: OR 0.77, CI 0.68–0.88; male sex: OR 0.03, CI 0.02–0.05). Being a sixth-year student was associated with a 3.5-fold increase in the likelihood of HPV vaccination uptake compared with first-year students (OR 3.45, CI 1.24–9.57). Furthermore, receiving an invitation for vaccination was positively associated with vaccination uptake (OR 1.70, CI 1.09–2.65). These findings were consistent with models considering both sexually active individuals and females. In the latter, a positive association was also observed for fifth-year students (OR 4.45, CI 1.24–16.11) (Figure 1).

When examining correlates with the percentage of knowledge, we observed significant negative associations with male sex and individuals with 2–5 sexual partners over the last year (respectively Coef: −1.29, CI −2.55 to −0.029 and − 2.23, CI −4.15 to −0.32). On the other hand, the year of enrollment displayed a positive likelihood of having a higher score (Coef: third: 4.86; fourth: 4.62; fifth: 8.50; sixth: 11.75; and for a further year: 7.51). The observed results, except for the number of sexual partners, were consistent for the female and sexually active populations (Table 2).

**Table 2:** Association between the knowledge percentage score and demographic characteristics, healthcare-related and organization factors, and sexuality.

Knowledge		Female population			Whole population			Sexually active population		
		Coef.	95%	CI	Coef.	95%	CI	Coef.	95%	CI
Sex	Female (ref)									
	Male				**−1.291**	**−2.552**	**−0.029**	**−1.665**	**−3.113**	**−0.217**
Age		−0.059	−0.533	0.415	0.152	−0.216	0.521	0.104	−0.284	0.492
Year of enrollment	1st (ref)									
	2nd	−0.112	−2.208	1.984	0.446	−1.234	2.126	0.778	−1.253	2.810
	3rd	**4.652**	**2.374**	**6.931**	**4.862**	**3.117**	**6.608**	**5.036**	**3.062**	**7.010**
	4th	**5.148**	**1.758**	**8.538**	**4.617**	**1.921**	**7.312**	**5.609**	**2.637**	**8.581**
	5th	**8.856**	**5.362**	**12.351**	**8.505**	**5.728**	**11.281**	**9.096**	**6.072**	**12.119**
	6th	**12.907**	**9.191**	**16.622**	**11.749**	**8.820**	**14.678**	**11.990**	**8.843**	**15.137**
	O.P.Y.	**9.297**	**4.025**	**14.568**	**7.512**	**3.573**	**11.451**	**8.169**	**4.027**	**12.311**
Smoking habits	No (ref)									
	Former-Smoker	−0.135	−3.190	2.919	0.192	−2.143	2.527	0.764	−1.658	3.187
	Yes	−0.061	−1.971	1.849	0.316	−1.200	1.832	0.485	−1.138	2.107
Province of residence	Napoli (ref)									
	Avellino and Benevento	−1.869	−5.327	1.588	−0.544	−3.148	2.060	1.296	−1.640	4.233
	Salerno	−0.072	−3.368	3.224	−0.747	−3.417	1.923	−1.048	−4.285	2.190
	Caserta	−0.415	−2.923	2.094	−0.674	−2.568	1.221	−0.100	−2.356	2.156
	Other	2.344	−0.882	5.571	1.408	−1.289	4.105	1.311	−1.583	4.205
HPV invitation	No/Don’t know (ref)									
	Yes	−0.995	−2.491	0.500	−0.769	−2.072	0.535	−0.852	−2.354	0.651
Number of sexual partners in the last year	0 (ref)									
	1	−1.522	−3.322	0.278	−1.205	−2.579	0.168	−0.918	−3.615	1.779
	2–5	−2.540	−5.088	0.008	**−2.237**	**−4.152**	**−0.322**	−2.086	−5.093	0.922
	6+	−6.277	−12.775	0.220	−2.778	−6.558	1.002	−2.571	−6.996	1.854
Gynecologist visit	Never (ref)									
	Rarely	−0.141	−2.073	1.791						
	Regularly	1.197	−0.808	3.202						
Condom use	No (ref)									
	Yes							0.865	−1.023	2.753

Multivariable linear regression models were employed for the whole population, female population, and sexually active population, including the knowledge percentage score as an outcome variable and controlled for the following variables: age, gender, year of enrollment, smoking habits, province of residency, received an invitation to undergo vaccination (HPV invitation), number of sexual partners over the last year, obi-gyn visit (only in the female population), and the use of condoms (only in the sexually active population). Results are presented as a regression coefficient (Coef) and 95% confidence intervals (95% CI). Bold values have been used to mark the statistically significant results.

The likelihood of having a higher attitude score was reduced by 62% for males (OR: 0.38, CI 0.29–0.50). Conversely, being in a higher year of enrollment had a positive correlation with the likelihood of having a higher score (OR: third: 1.87; fifth: 1.89; sixth: 2.16); these results were consistent when considering the female population, including the fourth year, and sexually active population, except for the fifth year. Only when considering the entire population, did former smokers (OR: 1.76) or individuals residing in the provinces of Avellino and Benevento (OR: 1.80) show an increased likelihood of having a higher attitude score (Table 3).

**Table 3:** Association between attitude score and demographic characteristics, healthcare-related and organization factors, and sexuality.

Attitude		Female population			Whole population			Sexually active population		
		OR	95%	CI	OR	95%	CI	OR	95%	CI
Sex	Female (ref)									
	Male				**0.380**	**0.287**	**0.504**	**0.430**	**0.312**	**0.592**
Age		1.025	0.926	1.134	1.015	0.938	1.098	1.010	0.929	1.098
Year of enrollment	1st (ref)									
	2nd	**1.705**	**1.083**	**2.686**	1.299	0.899	1.876	1.347	0.859	2.111
	3rd	**1.984**	**1.227**	**3.208**	**1.870**	**1.286**	**2.717**	**1.780**	**1.168**	**2.711**
	4th	**2.309**	**1.134**	**4.699**	1.750	0.991	3.092	1.641	0.872	3.087
	5th	**2.575**	**1.211**	**5.477**	**1.895**	**1.055**	**3.405**	1.661	0.884	3.120
	6th	**2.543**	**1.146**	**5.643**	**2.165**	**1.165**	**4.024**	**2.060**	**1.063**	**3.993**
	O.P.Y.	0.909	0.290	2.855	1.447	0.609	3.440	1.487	0.600	3.685
Smoking habits	No (ref)									
	Former-Smoker	1.163	0.604	2.240	**1.764**	**1.054**	**2.952**	1.682	0.995	2.844
	Yes	0.873	0.578	1.320	0.832	0.598	1.158	0.844	0.596	1.195
Province of residence	Napoli (ref)									
	Avellino and Benevento	1.493	0.726	3.070	**1.805**	**1.032**	**3.156**	1.583	0.847	2.959
	Salerno	0.811	0.409	1.610	0.908	0.523	1.575	0.820	0.429	1.568
	Caserta	1.188	0.704	2.003	1.144	0.772	1.696	1.179	0.734	1.894
	Other	0.917	0.482	1.747	1.105	0.628	1.946	1.033	0.562	1.899
HPV invitation	No/Don’tknow (ref)									
	Yes	1.261	0.917	1.733	1.119	0.847	1.479	1.199	0.869	1.653
Number of sexual partners over the last year	0 (ref)									
	1	1.083	0.740	1.584	1.224	0.916	1.636	1.308	0.741	2.307
	2–5	1.047	0.618	1.774	1.402	0.931	2.110	1.496	0.790	2.833
	6+	0.582	0.153	2.212	1.084	0.483	2.433	1.158	0.457	2.935
Gynecologist visit	Never (ref)									
	Rarely	1.152	0.769	1.726						
	Regularly	1.178	0.771	1.802						
Condom use	No (ref)									
	Yes							0.990	0.662	1.481

Multinomial logistic regression models were employed for the whole population, female population, and sexually active population, including the attitude score as an outcome variable and controlled for the following variables: age, gender, year of enrollment, smoking habits, province of residency, received an invitation to undergo vaccination (HPV invitation), number of sexual partners over the last year, obi-gyn visit (only in the female population), and the use of condoms (only in the sexually active population). Results are presented as odds ratios (OR) and 95% confidence intervals (95% CI). Bold values have been used to mark the statistically significant results.

### Sensitivity analysis

In our sensitivity analysis of the whole population, males showed a 97% reduction in the likelihood to be vaccinated against HPV (OR: 0.03, 95% CI: 0.02–0.05). For former smokers, the reduction was 61% (OR: 0.39, CI 0.17–0.92) compared with non-smokers, and for those resident in the province of Caserta, the reduction was 55% (OR: 0.45, CI 0.22–0.90) compared with those resident in the province of Napoli. Furthermore, increasing age showed a negative association with HPV vaccination (OR: 0.75, 95% CI: 0.66–0.86). The likelihood of being vaccinated was higher for those in the fifth or sixth year of enrollment (OR: respectively 3.53, 95% CI: 1.20–10.38 and 4.81, 95% CI: 1.59–13.51) compared with first-year students. We found a negative association with the percentage of knowledge (OR: 0.95, CI 0.93–0.98) and a positive association with attitude score (OR: 6.79, CI 3.91–11.79) (Figure 1). However, receiving an invitation for HPV vaccination did not show a significant association, even though the *p*-value was 0.051. These results were confirmed in the different samples, except for former smokers in the female population and for the fifth year of enrollment and residing in the province of Caserta in the sexually active population. Instead, the positive association with HPV invitation was found only in the sexually active sample (OR: 1.97, CI 1.12–3.46) (Table 4).

**Table 4:** Sensitivity analysis: association between HPV vaccination and demographic characteristics, healthcare-related and organization factors, sexuality, knowledge percentage score, and attitude score.

HPV vaccination		Female population			Whole population			Sexually active population		
Sensitivity analysis		OR	95%	CI	OR	95%	CI	OR	95%	CI
Sex	Female (ref)									
	Male				**0.030**	**0.018**	**0.050**	**0.027**	**0.015**	**0.049**
Age		**0.700**	**0.594**	**0.824**	**0.754**	**0.660**	**0.862**	**0.729**	**0.630**	**0.845**
Year of enrollment	1st (ref)									
	2nd	0.978	0.452	2.117	1.206	0.644	2.259	0.810	0.380	1.723
	3rd	1.350	0.592	3.080	1.089	0.572	2.072	0.865	0.409	1.831
	4th	2.168	0.607	7.739	1.814	0.661	4.980	1.650	0.527	5.167
	5th	**5.415**	**1.337**	**21.932**	**3.523**	**1.196**	**10.380**	2.853	0.872	9.334
	6th	**7.300**	**1.756**	**30.350**	**4.810**	**1.586**	**14.589**	**4.017**	**1.181**	**13.668**
	O.P.Y.	5.151	0.964	27.520	3.304	0.808	13.509	3.368	0.731	15.514
Smoking habits	No (ref)									
	Former-Smoker	0.457	0.157	1.328	**0.393**	**0.167**	**0.925**	**0.357**	**0.144**	**0.884**
	Yes	0.918	0.468	1.800	0.865	0.498	1.501	0.905	0.497	1.647
Province of residence	Napoli (ref)									
	Avellino and Benevento	4.650	0.495	43.724	2.206	0.809	6.015	2.749	0.892	8.471
	Salerno	0.471	0.170	1.303	0.456	0.189	1.100	0.387	0.129	1.155
	Caserta	**0.437**	**0.192**	**0.991**	**0.449**	**0.223**	**0.901**	0.626	0.268	1.460
	Other	0.690	0.224	2.126	2.251	0.788	6.432	2.120	0.684	6.570
HPV invitation	No/Don’tknow (ref)									
	Yes	1.732	0.991	3.027	1.607	0.998	2.586	**1.971**	**1.121**	**3.464**
Number of sexual partners over the last year	0 (ref)									
	1	1.110	0.583	2.113	1.202	0.725	1.992	0.817	0.309	2.161
	2–5	1.163	0.457	2.959	0.739	0.372	1.470	0.501	0.170	1.475
	6+	0.752	0.059	9.518	0.690	0.128	3.723	0.490	0.074	3.260
Gynecologist visit	Never (ref)									
	Rarely	1.371	0.688	2.731						
	Regularly	1.422	0.710	2.849						
Condom use	No (ref)									
	Yes							1.159	0.573	2.347
Attitude		**9.655**	**4.828**	**19.309**	**6.790**	**3.910**	**11.792**	**7.078**	**3.680**	**13.613**
Knowledge		**0.947**	**0.914**	**0.982**	**0.952**	**0.927**	**0.978**	**0.953**	**0.924**	**0.984**

Multivariate logistic regressions were employed for the whole population, female population, and sexually active population, including HPV vaccination as an outcome variable and controlled for the following variables: age, gender, year of enrollment, smoking habits, province of residency, received an invitation to undergo vaccination (HPV invitation), number of sexual partners over the last year, knowledge percentage score, attitude score, obi-gyn visit (only in the female population), and the use of condoms (only in the sexually active population). Results are presented as odds ratios (OR) and 95% confidence intervals (95% CI). Bold values have been used to mark the statistically significant results.

## Discussion

This survey-based cross-sectional study evaluated socio-demographic characteristics, healthcare-related and organization factors and barriers, and sexuality associated with knowledge, attitude, and self-reported HPV vaccination uptake in 882 medical students at the largest university in Southern Italy, from March to June of 2023. More than 60% of our study population were females, 78.5% of whom were vaccinated against HPV, as compared with 16.5% of males. We consistently observed that male sex and increasing age were associated with a lower likelihood of being vaccinated against HPV, whereas we found an increase in the likelihood of being vaccinated as students progressed in their academic career. Overall, these associations were confirmed when considering knowledge and attitude towards HPV. Additionally, we found evidence that active outreach from healthcare institutions was associated with a higher likelihood of receiving HPV vaccination.

Sex was a strong correlate across all analyses. This result has been described previously in literature, and official data (22, 23) which have highlighted sex-related inequalities in the availability of the HPV vaccination within Health Systems. However, the provision of free vaccination was made available to females long before it was extended to males, and for females this was accompanied by a catch-up strategy integrated within cervical cancer screening programs (24). Furthermore, HPV is perceived, due to its relationship with to cervical cancer, as a “female-only problem” (25).

Increasing age was associated with a lower likelihood of being vaccinated against HPV, as with each additional year, there was a 23% drop in the probability of being vaccinated. This evidence is supported by previous studies carried out in similar settings (26). A possible explanation for this result is the interest in the health system’s free-of-charge offer from the youngest subgroup of our study population (27).

Senior year students were associated with HPV vaccination uptake in both the entire and female populations, whereas no differences were observed in those who were sexually active. When restricting analyses to female students only, this association was further strengthened, and there was also a trend in fifth-year students. This finding has been reported in previous studies conducted in similar settings (28, 29). Although in our study, senior students were less represented, as the study was focused on students attending the first three years, a possible explanation tfor these results is that a growing level of academic knowledge is associated with the highest uptake (30). No differences were found regarding smoking habit, and although this correlation has not been broadly studied previously, it is in line with previous research (31).

Receiving an invitation increased the probability of being vaccinated against HPV. This evidence enlightens the role of an active call-in determining HPV vaccination uptake, and although it has been suggested by previous documents (32, 33), its quantification in terms of a direct effect on vaccination uptake is a novelty in the literature. Furthermore, no difference was found in the province of residency. No other study assessed this correlation, although this homogeneity may be due to HPV-related policies implemented at a regional rather than local health authority level (34).

We conducted our analysis in the largest university in Southern Italy, which accounts for over 4,000 medical students (approximately 4% of the medical students in Italy) (35). Hence, our results are likely to be representative of similar settings in the country. Framing determinants of HPV vaccination in medical students has provided insight into a population that, although sexually active, can be considered, at the same time, at low risk of HPV vaccination hesitancy due to the higher medical knowledge and education attainment. This information is pivotal to understanding the impact of targeted knowledge and attitude intervention on vaccine uptake. Nonetheless, some caveats merit discussion. First, recalling vaccination status and modality, on average, 10 years after adolescence, may have been difficult. Second, our sample was mostly represented by students attending the first 3 years of medical school, although the sex proportion was broadly comparable across years. Third, 2% of the study population did not complete the questionnaire. However, the number is very low and unlikely to have influenced our findings. Fourth, assessing vaccination history was challenging, as the vaccination uptake can primarily be a parental decision despite the under-25 catch-up strategies implemented by the national health system (36). Fifth, a potential selection bias may have resulted in more interested individuals participating. Nonetheless, the sample size was representative of the student population. Finally, another limitation of our study is that all participants were medical students. Although it would have been interesting to compare our population with non-medical students, we decided to focus on this specific population to assess whether the coverage had effectively increased in groups that are considered at low risk to further improve the public health strategy. However, such a comparison may be addressed in future studies.

### Policy

Medical students are an ideal population for identifying determinants of HPV vaccination uptake. A decrease in the rate of vaccination, exacerbated by the pandemic (37), may dramatically increase the incidence of HPV-related cancers, generating costs for the health system. Extending the free-of-charge offer for HPV vaccination to males is a cost-efficient measure for reducing the burden of cervical cancer (38); a tailored vaccination strategy targeted to the male population is mandatory to eradicate this infection. However, even in a population with high educational attainment and medical knowledge, we still found that HPV vaccination uptake was suboptimal, especially in men. Additionally, some age groups did not receive and were not eligible for a free-of-charge HPV vaccination (27), even though they were still considered at high risk due to other characteristics (e.g., sexual behavior). Therefore, additional public health interventions, including the extension of the recall strategy to men, need further implementation (39). In Italy, the new vaccination program “PNPV – 2023-25” in particular has extended the recall-free-of-charge offer (40); this implementation might further improve vaccination rates. However, the impact of the newly implemented national strategy cannot be evaluated yet. Furthermore, an effective and up-to-date communication strategy, using different and contemporary media, may impact strongly on the most resistant subgroups of the unvaccinated population (41). Finally, attitude and knowledge regarding HPV vaccination are strongly related to vaccination uptake (42, 43). Increasing awareness and knowledge in high-risk subgroups through tailored counseling strategies may raise vaccination rates (44).

## Conclusion

In this study, we found that HPV vaccination in medical students at University “Federico II” of Naples is higher than in the general population but still suboptimal. Sex and increasing age are strong drivers of vaccination uptake. We confirmed that an active call strategy, an important organizational factor, is essential for improving vaccination uptake. Further studies addressing the impact of other organizational factors that might influence HPV vaccination uptake should be conducted to better tailor interventions.

Our findings support the need to improve public health strategies to strengthen communication strategies and increase awareness and knowledge regarding HPV vaccination, even in hard-to-reach groups. A comprehensive strategy, which might also include extending the free-of-charge offer, may increase HPV vaccination uptake as well as improve the knowledge of and attitude toward vaccination in general.

## Data availability statement

The raw data supporting the conclusions of this article will be made available by the authors, without undue reservation.

## Ethics statement

The studies involving humans were approved by Ethical approval was granted by the University Hospital Ethics Committee (Prot. no. 0014525, 24th of March 2023). The studies were conducted in accordance with the local legislation and institutional requirements. The participants provided their written informed consent to participate in this study.

## Author contributions

MS: Data curation, Formal analysis, Methodology, Writing – original draft. MM: Writing – original draft. FE: Writing – original draft. AL: Writing – original draft. GB: Writing – original draft. CR: Writing – original draft. MT: Writing – original draft, Writing – review & editing. RP: Writing – original draft, Writing – review & editing.
