# Supplementary material for: Identification of organizational barriers to HPV vaccination uptake in medical students in southern Italy: a cross-sectional study
Source: Front Public Health. 2023 Nov 17;11:1272630. doi: 10.3389/fpubh.2023.1272630 (PMC10691465; doi:10.3389/fpubh.2023.1272630)
Supplement: Supplementary file 1 [file Table_1.docx]

| **HPV vaccination** | | **Female Population** | | | **Whole Population** | | | **Sexually Active Population** | | |
| --- | --- | --- | --- | --- | --- | --- | --- | --- | --- | --- |
| **Model I** | | **OR** | **95%** | **CI** | **OR** | **95%** | **CI** | **OR** | **95%** | **CI** |
| **Sex** | *Female (ref)* |  |  |  |  |  |  |  |  |  |
|  | *Male* |  |  |  | **0.029** | **0.019** | **0.044** | **0.026** | **0.016** | **0.043** |
| **Age** |  | **0.727** | **0.627** | **0.843** | **0.748** | **0.653** | **0.856** | **0.710** | **0.611** | **0.825** |
| **Year of enrollment** | *1st (ref)* |  |  |  |  |  |  |  |  |  |
|  | *2nd* | 1.210 | 0.615 | 2.381 | 1.244 | 0.703 | 2.199 | 0.805 | 0.401 | 1.616 |
|  | *3rd* | 1.569 | 0.775 | 3.176 | 1.351 | 0.743 | 2.455 | 1.019 | 0.510 | 2.034 |
|  | *4th* | **3.285** | **1.042** | **10.359** | 2.180 | 0.866 | 5.486 | 1.822 | 0.642 | 5.172 |
|  | *5th* | **5.219** | **1.504** | **18.108** | **2.975** | **1.137** | **7.785** | 2.551 | 0.880 | 7.396 |
|  | *6th* | **5.772** | **1.663** | **20.032** | **4.406** | **1.589** | **12.212** | **3.617** | **1.194** | **10.961** |
|  | *O.P.Y.* | 3.242 | 0.739 | 14.226 | 2.467 | 0.641 | 9.496 | 2.561 | 0.602 | 10.894 |
| **Smoking habits** | *No (ref)* |  |  |  |  |  |  |  |  |  |
|  | *Former-Smoker* | 0.597 | 0.253 | 1.413 | 0.553 | 0.257 | 1.187 | 0.492 | 0.220 | 1.097 |
|  | *Yes* | 0.816 | 0.458 | 1.457 | 0.774 | 0.471 | 1.270 | 0.757 | 0.438 | 1.309 |

**Table 1. Association between HPV vaccination and demographic characteristics.** For each of these populations: Whole Population, Female Population, and Sexually Active Population, multivariate logistic regressions were employed including HPV vaccination as outcome variable and controlled for the following variables: age, gender, year of enrollment, smoking habits. Results are presented as odds ratios (OR) and 95% confidence intervals (95% CI).

| **HPV vaccination** | | **Female Population** | | | **Whole Population** | | | **Sexually Active Population** | | |
| --- | --- | --- | --- | --- | --- | --- | --- | --- | --- | --- |
| **Model II** | | **OR** | **95%** | **CI** | **OR** | **95%** | **CI** | **OR** | **95%** | **CI** |
| **Sex** | *Female (ref)* |  |  |  |  |  |  |  |  |  |
|  | *Male* |  |  |  | **0.030** | **0.019** | **0.047** | **0.029** | **0.017** | **0.049** |
| **Age** |  | **0.727** | **0.622** | **0.849** | **0.757** | **0.660** | **0.869** | **0.719** | **0.616** | **0.838** |
| **Year of enrollment** | *1st (ref)* |  |  |  |  |  |  |  |  |  |
|  | *2nd* | 1.136 | 0.569 | 2.266 | 1.176 | 0.659 | 2.100 | 0.775 | 0.382 | 1.574 |
|  | *3rd* | 1.526 | 0.721 | 3.227 | 1.169 | 0.630 | 2.168 | 0.957 | 0.466 | 1.964 |
|  | *4th* | 2.461 | 0.769 | 7.874 | 1.875 | 0.726 | 4.844 | 1.641 | 0.561 | 4.802 |
|  | *5th* | **4.610** | **1.285** | **16.540** | **2.821** | **1.045** | **7.618** | 2.368 | 0.788 | 7.111 |
|  | *6th* | **5.617** | **1.554** | **20.307** | **3.818** | **1.355** | **10.756** | **3.194** | **1.030** | **9.908** |
|  | *O.P.Y.* | 3.257 | 0.694 | 15.298 | 2.463 | 0.615 | 9.861 | 2.732 | 0.607 | 12.295 |
| **Smoking habits** | *No (ref)* |  |  |  |  |  |  |  |  |  |
|  | *Former-Smoker* | 0.558 | 0.218 | 1.429 | 0.516 | 0.233 | 1.145 | 0.444 | 0.191 | 1.032 |
|  | *Yes* | 0.895 | 0.495 | 1.620 | 0.798 | 0.482 | 1.319 | 0.779 | 0.447 | 1.357 |
| **Province of residence** | *Napoli (ref)* |  |  |  |  |  |  |  |  |  |
|  | *Avellino and Benevento* | 5.491 | 0.641 | 47.066 | 2.439 | 0.950 | 6.263 | 2.822 | 0.961 | 8.288 |
|  | *Salerno* | 0.554 | 0.209 | 1.467 | 0.516 | 0.218 | 1.221 | 0.462 | 0.161 | 1.326 |
|  | *Caserta* | 0.574 | 0.267 | 1.237 | 0.554 | 0.290 | 1.058 | 0.716 | 0.327 | 1.570 |
|  | *Other* | 0.707 | 0.259 | 1.928 | 2.250 | 0.815 | 6.211 | 2.041 | 0.686 | 6.074 |
| **HPV invitation** | *No/Don'tknow (ref)* |  |  |  |  |  |  |  |  |  |
|  | *Yes* | **1.936** | **1.158** | **3.239** | **1.649** | **1.060** | **2.565** | **2.014** | **1.195** | **3.393** |
| **Ginecologist visit** | *Never (ref)* |  |  |  |  |  |  |  |  |  |
|  | *Rarely* | 1.367 | 0.730 | 2.561 |  |  |  |  |  |  |
|  | *Regularly* | 1.284 | 0.682 | 2.420 |  |  |  |  |  |  |

**Table 2. Association between HPV vaccination and demographic characteristics, and healthcare-related and organization factors.** For each of these populations: Whole Population, Female Population, and Sexually Active Population, multivariate logistic regressions were employed including HPV vaccination as outcome variable and controlled for the following variables: age, gender, year of enrollment, smoking habits, province of residency, having received an invitation to undergo vaccination (HPV invitation), and obi-gyn visit, only in Female Population. Results are presented as odds ratios (OR) and 95% confidence intervals (95% CI).

| **HPV vaccination** | | **Female Population** | | | **Whole Population** | | | **Sexually Active Population** | | |
| --- | --- | --- | --- | --- | --- | --- | --- | --- | --- | --- |
| **Model III** | | **OR** | **95%** | **CI** | **OR** | **95%** | **CI** | **OR** | **95%** | **CI** |
| **Sex** | *Female (ref)* |  |  |  |  |  |  |  |  |  |
|  | *Male* |  |  |  | **0.030** | **0.019** | **0.045** | **0.026** | **0.015** | **0.043** |
| **Age** |  | **0.723** | **0.623** | **0.840** | **0.741** | **0.647** | **0.850** | **0.708** | **0.608** | **0.825** |
| **Year of enrollment** | *1st (ref)* |  |  |  |  |  |  |  |  |  |
|  | *2nd* | 1.236 | 0.626 | 2.443 | 1.289 | 0.728 | 2.284 | 0.822 | 0.407 | 1.661 |
|  | *3rd* | 1.545 | 0.760 | 3.141 | 1.346 | 0.737 | 2.457 | 1.008 | 0.502 | 2.024 |
|  | *4th* | **3.339** | **1.054** | **10.584** | 2.290 | 0.901 | 5.821 | 1.844 | 0.644 | 5.276 |
|  | *5th* | **5.130** | **1.472** | **17.874** | **2.992** | **1.134** | **7.890** | 2.515 | 0.863 | 7.325 |
|  | *6th* | **5.741** | **1.641** | **20.084** | **4.390** | **1.569** | **12.282** | **3.656** | **1.182** | **11.305** |
|  | *O.P.Y.* | 3.207 | 0.725 | 14.181 | 2.500 | 0.642 | 9.735 | 2.607 | 0.599 | 11.342 |
| **Smoking habits** | *No (ref)* |  |  |  |  |  |  |  |  |  |
|  | *Former-Smoker* | 0.573 | 0.240 | 1.368 | 0.530 | 0.245 | 1.148 | 0.509 | 0.227 | 1.142 |
|  | *Yes* | 0.781 | 0.428 | 1.427 | 0.751 | 0.450 | 1.254 | 0.799 | 0.456 | 1.399 |
| **Number of sexual partners in the last year** | *0 (ref)* |  |  |  |  |  |  |  |  |  |
|  | *1* | 1.310 | 0.743 | 2.310 | 1.422 | 0.893 | 2.266 | 0.851 | 0.339 | 2.136 |
|  | *2-5* | 1.239 | 0.559 | 2.745 | 1.101 | 0.582 | 2.081 | 0.622 | 0.224 | 1.725 |
|  | *6+* | 1.260 | 0.139 | 11.437 | 1.106 | 0.256 | 4.775 | 0.685 | 0.127 | 3.685 |
| **Condom use** | *No (ref)* |  |  |  |  |  |  |  |  |  |
|  | *Yes* |  |  |  |  |  |  | 1.099 | 0.590 | 2.046 |

**Table 3. Association between HPV vaccination and demographic characteristics, and sexuality.** For each of these populations: Whole Population, Female Population, and Sexually Active Population, multivariate logistic regressions were employed including HPV vaccination as outcome variable and controlled for the following variables: age, gender, year of enrollment, smoking habits, number of sexual partners in the last year, and the use of condom, only in Sexually Active Population. Results are presented as odds ratios (OR) and 95% confidence intervals (95% CI).
