# Supplementary material for: Identification of organizational barriers to HPV vaccination uptake in medical students in southern Italy: a cross-sectional study
Source: Front Public Health. 2023 Nov 17;11:1272630. doi: 10.3389/fpubh.2023.1272630 (PMC10691465; doi:10.3389/fpubh.2023.1272630)
Supplement: Supplementary file 2 [file Data_Sheet_1.docx]

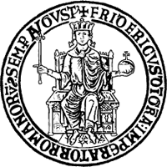


**UNIVERSITÀ DEGLI STUDI DI NAPOLI “FEDERICO II”**

**SCUOLA DI MEDICINA E CHIRURGIA DIPARTIMENTO DI SANITÀ PUBBLICA**

Questionnaire Code

**□□□□**

**ANONYMOUS QUESTIONNAIRE "ORGANIZATIONAL, MANAGERIAL AND COMMUNICATIVE BARRIERS FOR HPV VACCINATION"**

**Age** ____ years  **Sex: □** M □ F **Citizenship:** ________ **City of Residence**: __________ ( )

**Year of enrollment:** □ I □ II □ III □ IV □ V □ VI □ O.P.Y. **Do you smoke? □** Yes **□** No **□** Former-smoker

**C.d.L.: □** Medicine and Surgery **□** Technological Medicine **□** Dentistry **□** Health Professions **□** Nursing Sciences

| **You have had the HPV vaccination:** | □ Yes, at my ASL | □ Yes, at another ASL | □ Yes, by the pediatrician/general practitioner | □ Yes, at the gynaecologist |
| --- | --- | --- | --- | --- |
|  | □ Yes, other: | ___________ | □ No |  |
| **If yes, at what age? (in years)** | □ <15 | □ 15-19 | □ 20-25 | □ >25 |
| **Have you received an invitation to receive a free HPV vaccination?** | □ Yes, a letter | □ Yes, more than one letter | □ Yes, a phone call | □ I don’t know |
|  | □ Yes, more than a phone call | □ Yes, a phone call and a letter | □ Yes, more than one phone call and more than one letter | □ No |
|  | □ Yes, other | ___________ |  |  |
| **Have you had the HPV vaccination for a fee?** | □ Yes | □ No | □ I don’t know |  |
| **What suggestions did the family doctor/pediatrician (or other health care providers consulted) give you about this vaccination? (indicate only one answer)**   \| □ He/she/they Has/Have recommended vaccination. \| □ He/she/they advised against vaccination. \| \| --- \| --- \| \| □ He/she/they did not express an opinion on vaccination. \| □ He/she/they has/have advised to delay vaccination. \| \| □ Consulted more health, they expressed conflicting opinions. \| □ We have not spoken about. \| \| □ Other: ____ \|  \|   **Where did you get information about HPV/HPV vaccination? (more than one answer is possible**)   \| □I've never heard of HPV/HPV vaccination before. \| □Paediatrician/general practitioner  □Televisione/Radio \| □Operators of the vaccination service of the ASL \| \| --- \| --- \| --- \| \| □Operatori dei consultori \| □Gynecologist \| □Pharmacist \| \| □Family/Friends \| □School \| □Internet \| \| □Posters advertising/ flyers/ newspapers \| □Altro: ____ \|  \|   **If you have not been vaccinated at your ASL, specify the reason:** ____________________________________  **How much do you agree with the following statements from 1 to 5? 1= Not at all; 5= Completely agree** | | | | |

|  | **1** | **2** | **3** | **4** | **5** |
| --- | --- | --- | --- | --- | --- |
| HPV vaccination is not mandatory, so it is not important. | **□** | **□** | **□** | **□** | **□** |
| I have no confidence in vaccinations in general. | **□** | **□** | **□** | **□** | **□** |
| I believe that HPV infection is not serious. | **□** | **□** | **□** | **□** | **□** |
| I believe that this vaccination is not useful. | **□** | **□** | **□** | **□** | **□** |
| I am afraid of the side effects of this vaccination. | **□** | **□** | **□** | **□** | **□** |
| I haven't heard much about this vaccination and so I think it's not important.. | **□** | **□** | **□** | **□** | **□** |
| Vaccination is not important in males because they are not at risk. | **□** | **□** | **□** | **□** | **□** |
| Information received on HPV vaccination is conflicting. | **□** | **□** | **□** | **□** | **□** |
| Doctors advise against this vaccination. | **□** | **□** | **□** | **□** | **□** |
| Family/friends advise against this vaccination. | **□** | **□** | **□** | **□** | **□** |
| Those who are young and not sexually active do not need the vaccine. | **□** | **□** | **□** | **□** | **□** |
| I don't get vaccinated for religious reasons. | **□** | **□** | **□** | **□** | **□** |
| I wouldn't get vaccinated for a fee. | **□** | **□** | **□** | **□** | **□** |
| I use an alternative medicine that does not involve vaccinations. | **□** | **□** | **□** | **□** | **□** |
| It is difficult to meet the appointments made at the vaccination center. | **□** | **□** | **□** | **□** | **□** |
| It is not easy to reach the vaccination service. | **□** | **□** | **□** | **□** | **□** |
| It is complicated to book an appointment for vaccination. | **□** | **□** | **□** | **□** | **□** |

**Indicate true or false with reference to the following statements: True False**

| HPV infection is rare. | **□** | **□** |
| --- | --- | --- |
| HPV infection is transmitted through sexual intercourse. | **□** | **□** |
| HPV infection can cause cervical cancer. | **□** | **□** |
| Genital warts are caused by HPV. | **□** | **□** |
| HPV causes penile cancer. | **□** | **□** |
| HPV causes infertility. | **□** | **□** |
| Only women can be infected with HPV. | **□** | **□** |
| Most HPV infections regress spontaneously. | **□** | **□** |
| A person may be infected with HPV and not notice it. | **□** | **□** |
| Oral sexual intercourse is not at risk of HPV infection. | **□** | **□** |
| In some cases, HPV infection can last for many years.. | **□** | **□** |
| HPV vaccination works well when given before the onset of sexual activity. | **□** | **□** |
| HPV vaccination protects against all types of HPV that can cause cervical cancer. | **□** | **□** |
| Condoms protect against sexually transmitted diseases. | **□** | **□** |
| A high number of sexual partners increases the risk of HPV infection. | **□** | **□** |
| The onset of sexual activity at a young age increases the risk of HPV infection. | **□** | **□** |
| Having unprotected sex increases the risk of HPV infection. | **□** | **□** |
| The HPV vaccine is available for both sexes. | **□** | **□** |
| The HPV vaccine is effective even after the beginning of sexual life. | **□** | **□** |
| The HPV vaccine is mandatory in Italy for 12 years old girls. | **□** | **□** |
| The HPV vaccine is optional in Italy for 12 years old boys. | **□** | **□** |
| The HPV vaccine is free in Italy for girls and boys. | **□** | **□** |
| After the age of 12, you can no longer receive the HPV vaccine. | **□** | **□** |

**Ti ritieni sufficientemente informato su HPV/vaccinazione anti-HPV?** □ Yes □ No □ I don’t know

**You believe you may be at risk of HPV infection in the future?** □ Yes □ No □ I don’t know

**Are you planning to get vaccinated in the future? (e.g. other recommended vaccines, flu vaccine, etc...)**

□Yes, with all the vaccines offered by the doctor / vaccination service.

□Yes, with some of the proposed vaccines.

□No.

**You have carried out childhood vaccinations (against polio, diphtheria, tetanus, whooping cough, hepatitis B, etc.…)?**

| □ Yes, all those proposed by the pediatrician / vaccination service. | □ Yes, some of those proposed. |
| --- | --- |
| □ No. | □ I don’t know. |

**Would you recommend the HPV vaccine to your friends/relatives?** □ Yes. □ No. □ I don’t know.

**QHow many times do you go to the family doctor during the year?** □ 2 or more times □ Once □ Never □ Other__

**Fill in the following information about your parents:**

|  | Parent 1 | Parent 2 |
| --- | --- | --- |
| Sex: | M  F | M  F |
| Citizenship: | □ Italian  □ Other | □ Italian  □ Other |
| Age: | □ Under 35 years  □ Between 35 and 45 years  □ Between 45 and 55 years  □ More than 55 years | □ Under 35 years  □ Between 35 and 45 years  □ Between 45 and 55 years  □ More than 55 years |
| Educational qualifications: | □ Elementary school diploma  □ Media license  □ Diploma  □ Graduation | □ Elementary school diploma  □ Media license  □ Diploma  □ Graduation |
| Does She/He currently works? | □Yes □ No | □ Yes □ No |
| Lavora in ambiente sanitario? | □ Yes □ No | □ Yes □ No |
| What is she/he opinion about vaccinations in general? | □Favorable  □I don't know his/her opinion  □Against | □ Favorable  □I don't know his/her opinion  □ Against |
| Smoking habit: | □Non-smoker  □ Smoker  □Former-smoker | □ Non-smoker  □ Smoker  □Former-smoker |
| If female, how ofter she undergo a Pap-test: | □Yes, regularly  □Yes, rarely  □No  □I don't know | □Yes, regularly  □Yes, rarely  □No  □I don't know |

**Indicate your sexual orientation:** □ Heterosexual □ Homosexual □ Bisexual □ Other___

**Have you ever had sex?** □ Yes. □ No.

**If you answered the previous question positively:**

**At what age did you have your first sexual intercourse?** □ 12-14 years old □15-18 years old □ >19 years old

**Are you currently sexually active?** □ Yes □ No

**Contraceptive method used: (more than one answer is possible)**

| □ Condom | □ Female condom | □ Contraceptives | □ Morning-after pill | □ Spiral |
| --- | --- | --- | --- | --- |
| □ Diaphragm | □ Contraceptive patch | □ Vaginal ring | □ Coitus interrupted | □ Nobody |

**Have you been diagnosed with a sexually transmitted disease in the last year?** □ Yes □ No

**Number of sexual partners in the last year:** □ 1 □ From 2 to 5 □ More than 5

**How often have you used condoms in the last three months?**

| □ Never | □ Sometimes | □ Often | □ All the time |
| --- | --- | --- | --- |

**Have you ever had unprotected sex?**

| □ All the time | □ Yes, often | □ Yes, rarely | □ No |
| --- | --- | --- | --- |

**If woman:**

**Have you ever had a gynecological examination?** □ Yes, regularly □ Yes, rarely □ No

**Have you ever done a pap smear?** □ Yes, regularly □ Yes, rarely □ No

**If man:**

**Have you ever had a urological examination?** □ Yes, regularly □ Yes, once □ No
